# Canadian laboratory incidents with human pathogens and toxins: An overview of reports, 2016–2022

**DOI:** 10.14745/ccdr.v50i05a04

**Published:** 2024-05-24

**Authors:** Nathalie Balbontin, Audrey Gauthier, Christine Abalos, Antoinette N Davis, Meaghan Lister

**Affiliations:** 1 Regulatory, Operations and Emergency Management Branch, Public Health Agency of Canada, Ottawa, ON

**Keywords:** human pathogens and toxins, laboratory incidents, laboratory exposures, exposure severity, Laboratory Incident Notification Canada

## Abstract

**Background:**

When the Public Health Agency of Canada’s *Human Pathogens and Toxins Act* and *Human Pathogens and Toxins Regulations* came into force, the reporting of laboratory incidents to the Laboratory Incident Notification Canada (LINC) surveillance system became mandatory. This report summarizes the laboratory exposure and non-exposure data reported from 2016 to 2022, with a particular focus on factors that are not typically presented in LINC’s annual report.

**Methods:**

Reported laboratory incidents from 2016 to 2022 were analyzed. Exposures were analyzed by severity, occurrence and root cause, and affected individuals were analyzed by disease outcome, role and applied interventions. Non-exposures were analyzed by incident type. Exposure and non-exposure incident rates were calculated.

**Results:**

Events reported to LINC totalled 928. Of those, 355 were confirmed non-exposures, 361 were confirmed exposures, and 111 were other events. Both exposure and non-exposure incident rates per 100 active licences peaked in 2018 (9.44 and 7.11, respectively). Most exposures were rated as minor or negligible severity. The most cited exposure occurrence types were sharps-related and procedure-related (23% each), and standard operating procedure-related root causes were most cited (24%). While 781 individuals were affected in the exposure incidents, most did not develop a laboratory-acquired infection (n=753; 96%) and received at least one form of treatment post-exposure (n=717; 92%). Inadvertent possession/production cases were the most common non-exposure incidents reported.

**Conclusion:**

Exposure and non-exposure incident rates have decreased since 2018. Among exposure incidents, sharps-related and procedure-related occurrences were the most common, and the root cause was usually a standard operating procedure. Non-exposure incidents were mostly inadvertent possession/production cases. Exposure and illness outcome severity was mostly minor.

## Introduction

Human pathogens and toxins (HPTs) are routinely handled in laboratories for research purposes, as well as to detect and diagnose illnesses. Occasionally, individuals who work in laboratories are exposed to and infected by the HPTs they handle. These cases have been recorded worldwide and have highlighted the importance of biosafety and biosecurity measures ((1,2)).

The Public Health Agency of Canada (PHAC) promotes the safe handling of HPTs through the *Human Pathogens and Toxins Act* (HPTA) and the *Human Pathogens and Toxins Regulations* (HPTR). Under the HPTA and HPTR, any laboratory conducting activities within the scope of the HPTA must be licensed to do so, and licence holders are required to report laboratory incidents to PHAC (3,4).

Laboratory Incident Notification Canada (LINC) was launched in December 2015 as a comprehensive surveillance system that would receive incident reports required by the HPTA and the HPTR. Situations reported to LINC can generally be grouped into three categories: exposure incidents, non-exposure incidents, and other events requiring notification. Exposure incidents are incidents where one or more individuals have “contact with, or close proximity to, infectious material or toxins that may result in infection or intoxication, respectively” ((5)). This includes cases where exposure leads to a laboratory-acquired infection (LAI). Non-exposure incidents include the inadvertent possession, production, or release of an HPT that one is not licensed to work with. Instances where HPTs are missing, lost or stolen are also categorized as non-exposure incidents ((5)). Finally, as an example of an “other event requiring notification,” licensed parties must report upcoming changes to the laboratory that could affect biocontainment ((5)).

Laboratory Incident Notification Canada publishes annual reports that describe the laboratory incidents that occurred each year ((6–12)) to raise awareness about laboratory safety and highlight important information on laboratory exposures in Canada. These reports focus on exposure incidents and present incidents by main activity being performed at the time of the exposure incident and by sector (e.g., academia, government, industry). Information regarding the biological agent(s) involved, root cause(s) of the exposures and affected individuals (main role, years of experience, route of exposure) is also provided, along with reporting delay times and exposure incident rates.

The objective of this article is to analyze all relevant laboratory incidents reported to LINC between 2016 and 2022, examine factors not typically presented in the annual reports (e.g., the severity of incidents and interventions for exposed individuals), and discuss year-to-year trends.

## Methods

Exposure incidents, non-exposure incidents, and other events requiring notification are reported through PHAC’s Biosecurity Portal using standardized forms. The choice of form depends on the type of event being reported, with distinct forms available for each category. Each form includes a set list of questions for the reporter to answer; most questions are mandatory and closed-ended. Entered data is captured via the Microsoft Customer Relationship Management system and reviewed for consistency and completeness by LINC employees.

The LINC surveillance data was extracted to Microsoft Excel on August 8, 2023, and then processed and analyzed using R 4.2.1. Given that, more than one report can be submitted for a single incident if the reporter has information to add or correct. When multiple records for the same incident were submitted, only the most recent data was retained.

Reports about incidents that are outside the scope of the HPTA are sometimes submitted to LINC. For example, the HPTA does not regulate activities that involve Risk Group 1 (RG1) agents, nor does it require incidents with RG1 agents to be reported. These types of reports are stored by LINC but are often incomplete because they are not mandatory. Consequently, these reports have been excluded from analysis and are referred to as “ruled out.” Affected individuals were ruled out if the event itself was ruled out or if the individual was otherwise determined not to be exposed. This study focuses on incidents that involved Risk Group 2, 3 and 4 (RG2, RG3 and RG4, respectively) agents, which must be reported to LINC under the scope of HPTA.

Data from incidents that occurred between January 1, 2016, and December 31, 2022, was used in this analysis. Reports with an unknown incident date that were reported during this period were included. The severity, occurrence types, and root causes of exposure incidents were examined. Data on affected individuals, such as illness presentation, roles and treatments received, was also examined.

Data is continuously updated as LINC receives more information on incidents. Therefore, there may be minor discrepancies between the values published in LINC’s annual reports and those in this report (e.g., the total number of exposure incidents in a given year).

## Results

**Figure 1:**
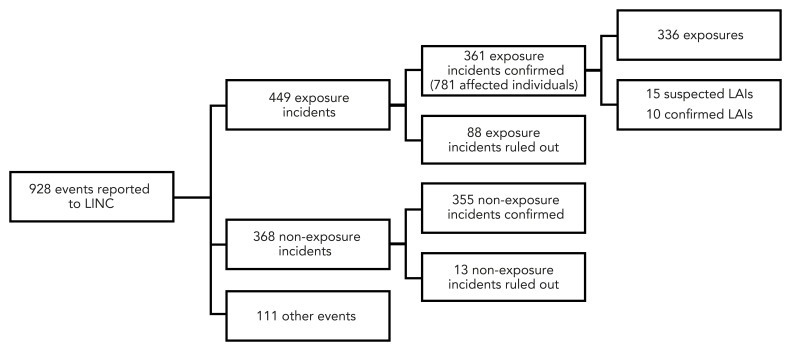
Types of events reported to Laboratory Incident Notification Canada, Canada, 2016–2022 Abbreviations: LAIs, laboratory-acquired infections; LINC, Laboratory Incident Notification Canada

Between January 1, 2016, and December 31, 2022, 928 events were reported to LINC. After investigation, 88 exposure incidents and 13 non-exposure incidents were ruled out. The following were retained: 361 exposure incidents, 355 non-exposure incidents, and 111 other events requiring notification (**Figure 1**). Among the 361 confirmed exposure incidents, 15 were suspected LAIs and 10 were confirmed LAIs. These LAIs are described in detail in another publication ((13)). While 819 persons were initially reported as being exposed in the 361 laboratory exposure incidents, 38 persons were ruled out, bringing the total to 781 exposed people between 2016 and 2022.

**Table 1** provides a comprehensive overview of how many laboratory incidents were reported from 2016 to 2022. For context, the number of active licences steadily increased over these seven years, rising from 835 in 2016 to 1,048 in 2022. Conversely, the number of exposure incidents demonstrated some fluctuations, with the highest count recorded in 2018 (93 incidents) and the lowest in 2020 and 2022 (both 39 incidents). The number of non-exposure incidents showed some variation as well, with the highest count in 2018 (70 incidents) and the lowest in 2020 (27 incidents). Year-over-year trends show that 2018 experienced a notable increase in both exposure and non-exposure incidents, while 2020 marked a significant decrease in these incidents.

**Table 1:** Number of confirmed exposure and non-exposure incidents and respective incident rates, Canada, 2016–2022

Year	Number of active licences	Number of exposure incidents	Number of non-exposure incidents	Exposure incidents per 100 active licences	Non-exposure incidents per 100 active licences
2016	835	43	51	5.15	6.11
2017	905	42	63	4.64	6.96
2018	985	93	70	9.44	7.11
2019	996	61	63	6.12	6.33
2020	999	39	27	3.90	2.70
2021	1,027	44	33	4.28	3.21
2022	1,048	39	48	3.72	4.58

**Figure 2:**
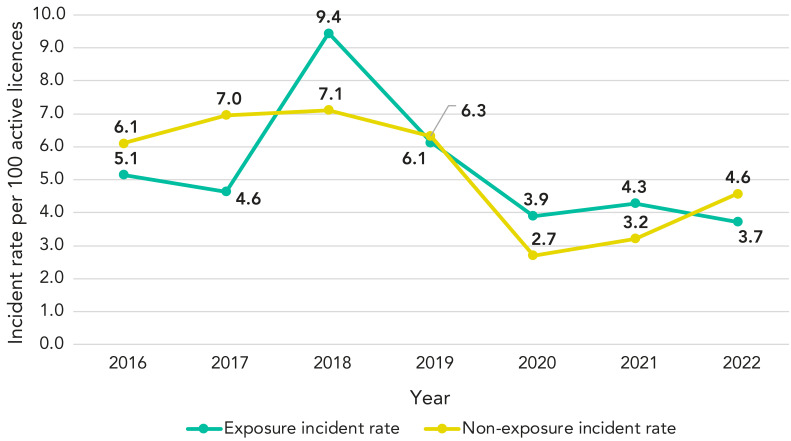
Exposure and non-exposure incident rates, Canada, 2016–2022

When examining the proportion of exposure incidents per 100 active licences, 2018 stood out as the year with the highest rate (9.44), while 2022 had the lowest (3.72), as shown in **Figure 2**. The proportion of non-exposure incidents per 100 active licences also peaked in 2018 (7.11) and reached its lowest point in 2020 (2.72).

### Severity of exposure incidents

**Figure 3:**
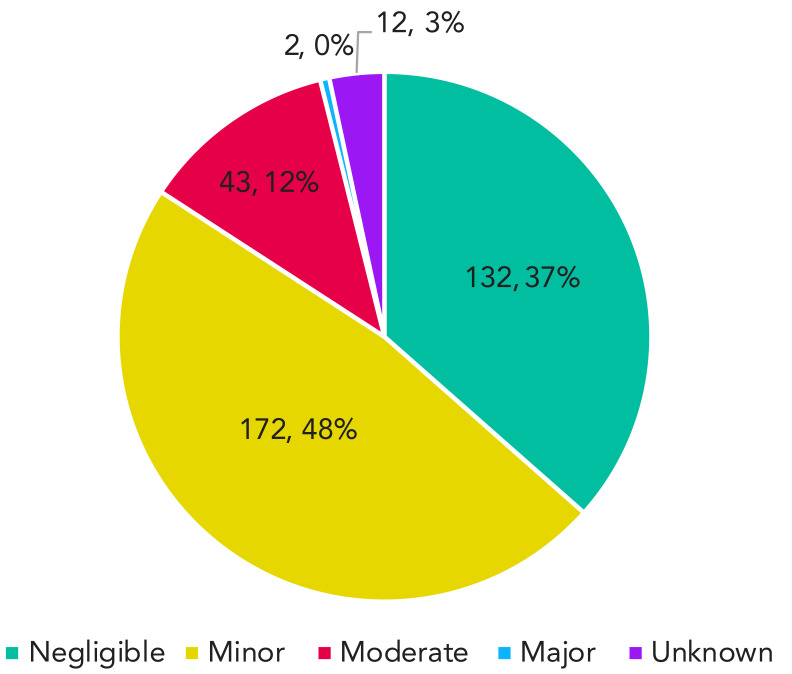
Total reported severity of exposure incidents, Canada, 2016–2022

As part of the investigation process for exposure incidents, reporters are asked to assess the severity of incidents. Reporters must provide a subjective rating that is based on the incident’s impact on individuals, other staff, and public health. Definitions for each level of severity are provided in **Appendix,** **Table A1**. Among the 361 exposure incidents reported between 2016 and 2022, 84% were either negligible or minor in severity (**Figure 3**). Exposure incidents of minor severity accounted for 48% (n=172) of incidents, while incidents of negligible severity accounted for 37% (n=132) of incidents. Only two incidents (0.01%) were classified as majorly severe; both involved a suspected LAI. No exposure incident was classified as catastrophic, which is the highest level of severity.

The two major incidents occurred in 2017 and 2019. The first incident was an exposure by inhalation of *Mycobacterium* spp. in a histology laboratory. The affected individual received the appropriate post-exposure treatment. The source of the second incident was not confirmed; it could not be determined whether the infection was acquired in the laboratory or through a community outbreak in the worker’s region. For both incidents, measures were taken to mitigate the risk of reoccurrence, including additional training and decontamination of laboratory areas.

**Figure 4:**
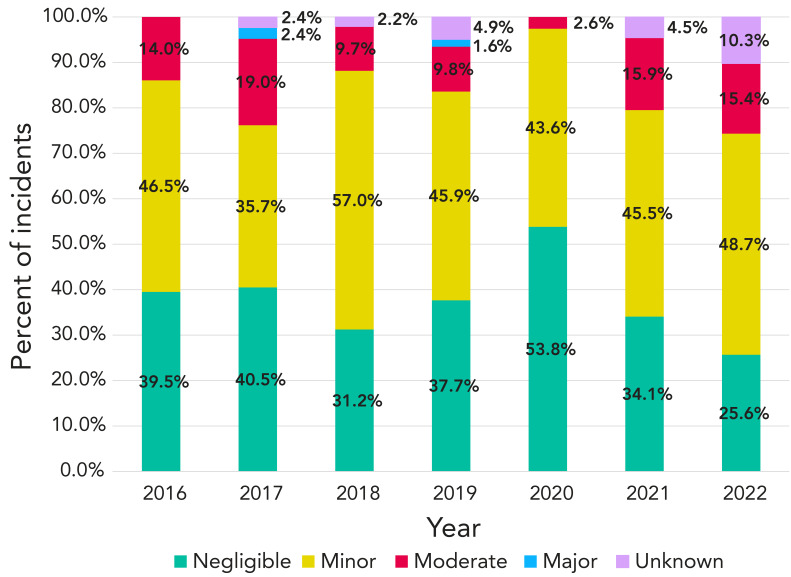
Reported severity of exposure incidents each year, Canada, 2016–2022

While similarities in the proportions of severity of incidents were observed every year from 2016 to 2022 (**Figure 4**), there were some differences. Most notably, there was a higher proportion of minor incidents in 2018 (57%), and 2020 marked the highest proportion of negligible incidents (54%) and the lowest proportion of moderate incidents (3%).

### Occurrence types for exposure incidents

When submitting an exposure report, reporters must select one or more occurrence types that best characterize the incident. **Table 2** presents the percentage of citations for each occurrence type out of the total number of occurrence types cited in all exposure events. Sharps-related and procedure-related issues were the most cited occurrence types overall. Definitions for occurrence types are found in Appendix, **Table A2**.

**Table 2:** Reported occurrence types of exposure incidents, Canada, 2016–2022

Occurrence type	Percentage of total occurrence types each year															
	2016		2017		2018		2019		2020		2021		2022		2016–2022	
	(N=62)		(N=56)		(N=118)		(N=79)		(N=55)		(N=56)		(N=60)		(N=486)	
	n	%	n	%	n	%	n	%	n	%	n	%	n	%	n	%
Animal-related	7	11	1	2	8	7	1	1	0	0	8	14	5	8	30	6
Equipment-related	1	2	1	2	8	7	6	8	6	11	3	5	1	2	26	5
Loss of containment	2	3	4	7	8	7	3	4	1	2	2	4	2	3	22	5
Other	7	11	9	16	11	9	12	15	5	9	6	11	6	10	56	12
PPE-related	10	16	6	11	12	10	8	10	8	15	10	18	8	13	62	13
Procedure-related	13	21	12	21	27	23	18	23	16	29	10	18	15	25	111	23
Sharps-related	14	23	13	23	28	24	16	20	13	24	12	21	15	25	111	23
Spill	5	8	8	14	14	12	11	14	6	11	2	4	5	8	51	10
Unknown	3	5	2	4	2	2	4	5	0	0	3	5	3	5	17	4

Abbreviation: PPE, personal protective equipment

Note: The total percentage for each column does not necessarily add up to 100% due to the rounding of numbers in the table

### Root causes of exposure incidents

When carrying out an investigation following an exposure incident, one or more root causes can be cited in the exposure follow-up report. **Table 3** shows the percentage of root causes for each year. From 2016 to 2022, 863 root causes were cited in the 361 exposure incidents. Overall, the most cited root causes were related to standard operating procedures (n=211, 24%), human factors (n=183, 21%) and equipment (n=114, 13%). Through the years, human factors were increasingly cited as a root cause (+1.46 citations per year) while there was a decrease in citations related to standard operating procedures (−3.29 citations per year) and other root causes (−2.36 citations per year). Examples of each type of root cause can be found in Appendix, **Table A3**.

**Table 3:** Reported root causes of exposure incidents, Canada, 2016–2022

Root cause	Citations as a percentage of total root causes each year															
	2016		2017		2018		2019		2020		2021		2022		2016–2022	
	(N=92)		(N=97)		(N=237)		(N=145)		(N=99)		(N=109)		(N=84)		(N=863)	
	n	%	n	%	n	%	n	%	n	%	n	%	n	%	n	%
Communication	10	11	10	10	25	11	17	12	9	9	11	10	7	8	89	10
Equipment	7	8	11	11	32	14	20	14	13	13	17	16	14	17	114	13
Human factors	8	9	13	13	53	22	35	24	24	24	30	28	20	24	183	21
Management and oversight	11	12	7	7	25	11	21	14	11	11	11	10	10	12	96	11
SOP	31	34	35	36	53	22	27	19	25	25	21	19	19	23	211	24
Training	7	8	8	8	27	11	17	12	10	10	15	14	7	8	91	11
Other	18	20	13	13	22	9	8	6	7	7	4	4	7	8	79	9

Abbreviation: SOP, standard operating procedure

Note: The total percentage of root causes for each year does not necessarily add up to 100% due to the rounding of numbers in the table

### Affected individuals

Between 2016 and 2022, 781 individuals were exposed to an HPT, which is an average of 2.16 affected individuals per exposure incident. Most exposed individuals (n=753, 96%) did not go on to develop a LAI. For exposed individuals, 2% (n=17) were suspected of having a LAI, while 1% (n=8) had a confirmed LAI. Less than 1% (n=3) were reported as having seroconversion.

Of those that experienced acute illness (n=23, 3%), six individuals recovered within a week, 11 recovered within one to two weeks, and three individuals recovered after the two-week mark. Recovery time remains unknown for three individuals. No report of chronic illness resulting from a laboratory exposure was received between 2016 and 2022.

**Figure 5:**
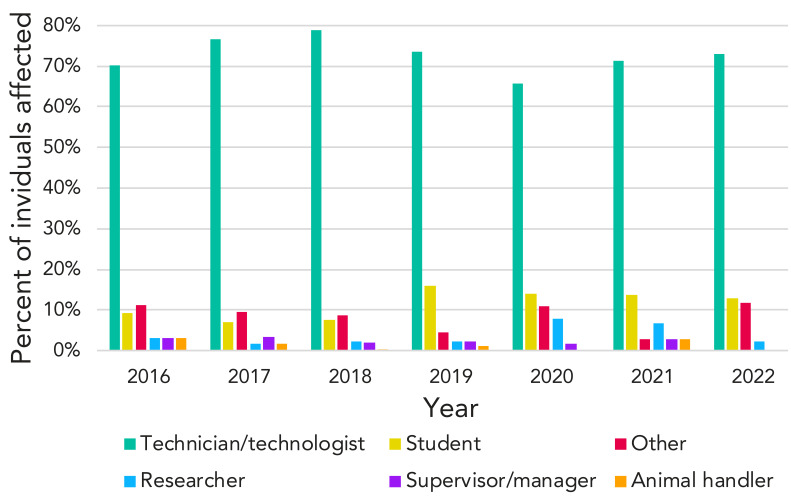
Role of affected individuals in exposure incidents each year, Canada, 2016–2022

Among all years, the most common role of exposed individuals was that of a technician/technologist (**Figure 5**). They represented 74% of all exposed individuals between 2016 and 2022 (n=581). Students represented the second-largest group of exposed individuals (10% of all exposed individuals).

Multiple choices of interventions can be selected for each affected individual. Of the 781 individuals who were exposed, 8% (n=64) did not receive any treatment or participate in a health consultation. For the remaining 92% (n=717), at least one form of intervention was employed. The average number of interventions employed per exposed individual was 2.2 interventions. As shown in **Table 4**, the most common intervention was an occupational health consultation within seven days of exposure. For affected individuals, 31% (n=245) not only received an intervention within seven days of exposure, but also received an intervention beyond seven days of exposure (data not shown on the table).

**Table 4:** Interventions for exposed individuals, Canada, 2016–2022

Interventions employed	n^a^	%
**Within 7 days of exposure**	**644**	**82%**
OH consultation	513	66%
Medical consultation	389	50%
PEP	215	28%
First-aid	138	18%
**Beyond 7 days of exposure**	**318**	**41%**
OH consultation	200	26%
Medical consultation	159	20%
Drug treatment	53	7%
PEP	46	6%

Abbreviations: OH, occupational health; PEP, post-exposure prophylaxis

^a^ Multiple options can be selected for one exposed individual

Note: The denominator of the percentage calculation for each intervention employed is the total number of affected individuals (781 individuals)

### Non-exposure incidents

**Figure 6:**
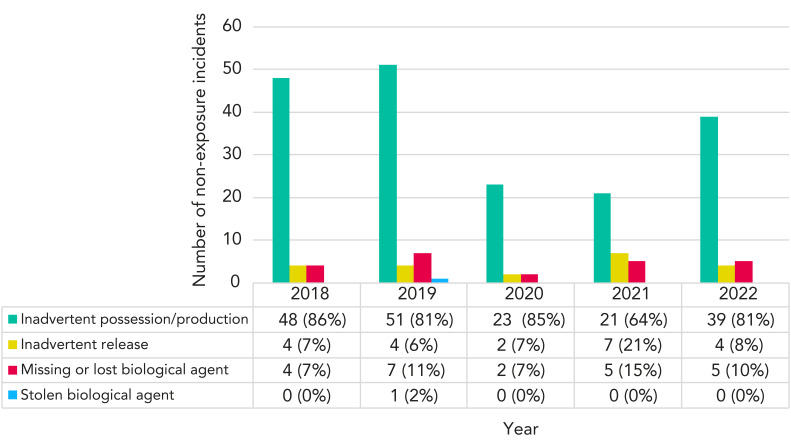
Types of non-exposure incidents, Canada, 2018–2022

Since March 2018, reporters have been able to specify what type of non-exposure incident they are reporting (**Figure 6**). Between 2018 and 2022, most non-exposure incidents involved the inadvertent possession/production of an HPT (ranging from 64% to 86%). In 2021, the proportion of reports involving inadvertent release (21%) and missing or lost biological agents (15%) peaked. LINC received only one report of a stolen biological agent in 2019. The report was filed by the institution following a student’s threat to steal a biological agent. When PHAC followed up with the reporter for more details, it was determined that no theft had occurred, and the incident was subsequently ruled out.

## Discussion

Over the past seven years of operation, LINC has received 361 confirmed exposure incidents and 355 non-exposure incidents ((6–12)). During that time, 781 individuals were exposed to an HPT, and 25 of those individuals developed, or were suspected to have developed, an LAI. According to reporters, most exposure incidents posed a low individual and public health risk. This is supported by the fact that most individuals did not experience any illness following exposure to an HPT. Individuals who did experience illness recovered within a couple of weeks.

### Changes in root causes of exposure incidents

In the investigatory process after an exposure incident occurs, reporters are asked for the plausible root cause(s) of the incident. One or more root causes can be selected. The number of citations reporting standard operating procedures as a root cause as a proportion of all cited root causes of exposure incidents decreased from 2016 to 2022. This decline may be a result of LINC’s investigatory process. When an exposure report is submitted to LINC, root causes and corrective actions are established to prevent similar incidents from reoccurring. This process may have prompted licensed facilities to refine their standard operating procedures, thus reducing the number of incidents caused by poor or missing documentation.

A root cause of incidents that increased in the number of citations every year relative to 2016 involved human factors. According to the exposure reporting form, human factors include decisions made by individuals working directly with HPTs (e.g., deviating from a standard operating procedure) and decisions made by other individuals who influence the work environment (e.g., managers who provide insufficient time to do a job safely). From 2016 to 2022, the proportion of all root causes that cited human factors increased from around 10% of citations at the beginning of the program, to around 25% in later years. This increase may be explained by changes in the exposure reporting form. In March 2018, several multiple-selection questions were added to the form and these questions provided specific examples of human factors. The clarification of human factors as a root cause may have helped reporters recognize their role in the incidents they were reporting. In fact, the greatest year-to-year increase in reports citing human factors was observed in 2018, which was the year the form was amended.

### Interventions for individuals exposed to human pathogens and toxins

Exposure to HPTs when conducting controlled experiments poses the risk of acquiring an infection. Appropriate post-exposure follow-up on the exposed individual can prevent or mitigate the severity of disease. While the mandated reporting of LAIs is unique to Canada, the outcomes of affected individuals in other countries has been captured through case studies. Case studies have suggested that timely administration of post-exposure prophylaxis can minimize or prevent infections. When administered early, post-exposure prophylaxis has been shown to be effective in preventing the acquisition of disease in high-risk individuals ((14)).

When conducting controlled activities, the correct implementation of nationally and internationally certified protocols with proper microbiological practices, containment devices, satisfactory facilities, protective barriers and specialized education and training may decrease the risk of exposure of laboratory staff to acquiring a laboratory infection ((15)). Details on post-exposure interventions for the affected individuals are collected by LINC. Of the reported exposure incidents in Canada, 82% of the affected individuals received a medical intervention within seven days of exposure, meaning that actions were taken to assess the health of the individual, and that appropriate health measures were taken for the majority of affected individuals.

### Exposure incidents of negligible to minor severity

Results showed that most exposure incidents (n=304, 84%) for the period of interest had a negligible to minor level of severity, which represents a low to minimal risk for disease in the individual and other staff members, as well as low or no risk to public health. A moderate level of severity was reported for 12% (n=43) of all exposure incidents, representing a moderate risk for the individual, the employee and public health. Only 1% (n=2) of all the exposure incidents were reported at a major severity level, representing a high risk of disease in the individual or employee and a significant risk to public health. The trend in severity of exposure incidents every year was similar from 2016 to 2022. It should be noted that the severity level is self-assessed by the reporter on site.

The fact that the majority of incidents had a negligible to minor level of severity suggests that laboratory procedures and safety measures seem highly effective in preventing major and catastrophic laboratory incidents. Several factors can contribute to the biosafety of staff members in licensed laboratories in Canada, such as preventive strategies that help mitigate the risk associated with working with human pathogens and toxins in laboratory settings. All biosafety measures in place in the laboratory, including proper training, use of personal protective equipment, and standard operating procedures play an important role in protecting laboratory staff members and reducing the risk of exposure incidents. The ongoing training of laboratory employees is also essential to gain the necessary awareness of safety in handling biohazardous materials ((15)). PHAC also plays a key role in the response, support, and information sharing needed to improve biosafety standards through its laboratory incident surveillance program.

Awareness of general biosafety measures has also increased during the SARS-CoV-2 pandemic ((16)). The Government of Canada’s *Canadian Biosafety Standard, Third Edition* is the national standard for facilities conducting activities with human pathogens and toxins. This document outlines the minimum physical containment, operational practice, and performance and verification testing requirements for facilities where RG2, RG3 and RG4 human or terrestrial animal pathogens or toxins are handled and stored ((17)).

## Limitations

The main strength of this study is that it is based on the mandatory and standardized reporting of laboratory incidents across Canada. All reports are reviewed by LINC employees and data can be updated if needed. Furthermore, LINC has been continuously collecting data since its inception in December 2015, which allows for an understanding of how Canada’s laboratory biosecurity landscape has changed over time.

A limitation of this study is that potential under-reporting of exposure and non-exposure incidents can make the data incomplete. To date, LINC has not been able to establish the extent of under-reporting. However, PHAC carries out regular inspections of licensed facilities to verify compliance with *Canadian Biosafety Standards* ((17)). One requirement of this standard is to keep an internal record of all biosafety and biosecurity incidents. During a laboratory inspection, a cross-reference check is carried out to ensure that internally recorded incidents were reported to PHAC. It is also important to consider that the data is self-reported and that measures like incident severity are based on the judgment of the reporter. The reporter may not be directly involved in the incident and must rely on another individual’s account of what occurred. Additionally, there are limitations to the calculations that involve the number of active licences. These calculations are done with the final number of licences active at the end of the given year. This does not give an accurate picture of active licence number fluctuations, as licence additions and revocations occur throughout the year.

No explicit explanation exists for the increase in the number of exposure incidents in 2018. It remains unknown whether the increase was due to an actual increase in incidents or in incident reporting. It is possible that as the surveillance program became more established, there was increased awareness of the need to report and the importance of doing so, which may have resulted in increased reporting.

Though data on international instances of LAIs have been captured through surveys ((2)), PHAC’s surveillance system provides a comprehensive mandated reporting system that operates at the national level to collect and analyze data on all laboratory incidents that involve HPTs. However, due to the lack of systematic worldwide reporting, it is difficult to compare Canadian laboratory incident data with that of other countries.

It should also be noted that although the percentage of citations for each occurrence type out of the total number of occurrence types cited for all exposure events was presented, certain occurrences, such as those involving animals, can only happen in facilities that work with animals, while other occurrences (i.e., procedure-related) have the potential to occur in all facilities.

Finally, the full impact of the coronavirus disease 2019 (COVID-19) pandemic on laboratory incident reporting and trends is not yet known. While many laboratories were actively involved in COVID-19 testing and research, laboratories also faced closures and reduced on-site staffing to mitigate virus transmission among employees. These changes in laboratory activity may have influenced the exposure and non-exposure incident rates from 2020 to 2022.

## Conclusion

Between 2016 and 2022, the exposure incident rate decreased over time, reaching its lowest point of 3.7 exposure incidents per 100 active licences in 2022. Year after year, the number of non-exposure reports followed a similar trend, with the majority of report types being inadvertent possession or production. The severity of the laboratory exposure incidents was mostly reported as negligible and minor. The most cited occurrence types were sharps-related, spills, and procedure-related. An overall increase in human factors and a decrease in standard operating procedures as a cited root cause was observed. Affected individuals, mostly technicians or technologists, rarely developed an illness.

The increased awareness of safe laboratory practices is integral to reducing biohazardous risk in these settings. The LINC surveillance system program will continue to provide oversight and disseminate laboratory incident information to the public and to licensed laboratories to increase awareness of risks when working with HPTs.

## Appendix

**Table A1:** Levels of severity for exposure incidents

Level of severity	Definition
Negligible	Minimal risk for disease in the individual/other staff AND no risk to public health
Minor	Low risk for disease in the individual/other staff and/or low risk to public health
Moderate	Moderate risk for disease in the individual/other staff and/or moderate risk to public health (limited spread among close contacts, no deaths)
Major	High risk of severe disease/death in the individual/other staff and/or significant public health impact (community spread/outbreak/fatalities)
Catastrophic	High risk of severe disease in the individual/other staff AND severe public health impact (severe epidemic/high mortality, etc.)

**Table A2:** Definitions of occurrence types

Occurrence type	Definition
Spill	Any unintended release of an agent from its container
Loss of containment	Includes malfunction or misuse of containment devices or equipment and other types of failures that result in the agent being spilled outside of or released from containment
Sharps-related	Needle stick, cut with scalpel or blade, or other sharps injury (i.e., broken glass)
Animal-related	Includes animal bites or scratches, as well as other exposure incidents resulting from animal behaviour (i.e., animal movement resulting in a needle stick)
PPE-related	Includes either inadequate PPE for the activity or failure of the PPE in some way
Equipment-related	Includes failure of equipment, incorrect equipment for the activity, or misuse of equipment
Procedure-related	Includes instances when written procedures were not followed, were inadequate or absent, or were incorrect for the activity

Abbreviation: PPE, personal protective equipment

**Table A3:** Root causes and examples

Root cause	Examples
Human factors	A violation (cutting a corner, not following correct procedure, deviating from standard operating procedure) An error (a mistake, lapse of concentration, or slip of any kind)
Standard operating procedure	Documents were followed as written but were not correct for activity/task Procedures that should have been in place were not in place Documents were not followed correctly
Equipment	Equipment quality control needed improvement Equipment failed Equipment was not appropriate for purpose
Training	Training not in place but should have been in place Training not appropriate for task/activity Staff were not qualified or proficient in performing task
Communication	Communication did not occur but should have Communication was unclear, ambiguous, etc.
Management and oversight	Supervision needed improvement Lack of auditing of standards, policies and procedures Risk assessment needed improvement
Other	Not applicable

## References

[1] Pike RM. Laboratory-associated infections: incidence, fatalities, causes, and prevention. Annu Rev Microbiol 1979;33(1):41–66. 10.1146/annurev.mi.33.100179.000353 386929

[2] Wurtz N, Papa A, Hukic M, Di Caro A, Leparc-Goffart I, Leroy E, Landini MP, Sekeyova Z, Dumler JS, Bădescu D, Busquets N, Calistri A, Parolin C, Palù G, Christova I, Maurin M, La Scola B, Raoult D. Survey of laboratory-acquired infections around the world in biosafety level 3 and 4 laboratories. Eur J Clin Microbiol Infect Dis 2016;35(8):1247–58. 10.1007/s10096-016-2657-1 27234593 PMC7088173

[3] Government of Canada. Human Pathogens and Toxins Act. Ottawa, ON: GoC. [Accessed 2024 April 19]. https://lois-laws.justice.gc.ca/eng/acts/H-5.67/FullText.html

[4] Government of Canada. Human Pathogens and Toxins Regulations. Ottawa, ON: GoC. [Accessed 2024 April 19]. https://gazette.gc.ca/rp-pr/p2/2015/2015-03-11/html/sor-dors44-eng.html

[5] Government of Canada. Notification and Reporting under the HPTA and HPTR using the Reporting Module of the Biosecurity Portal. Ottawa, ON: GoC. [Accessed 2024 April 19]. https://www.canada.ca/en/public-health/services/canadian-biosafety-standards-guidelines/guidance/notification-reporting-human-pathogens-toxins-act-regulations.html

[6] Abalos C, Gauthier A, Davis A, Ellis C, Balbontin N, Kapur A, Bonti-Ankomah S. Surveillance of laboratory exposures to human pathogens and toxins, Canada, 2022. Can Commun Dis Rep 2023;49(9):398–405. 10.14745/ccdr.v49i09a06 38463906 PMC10919944

[7] Thompson ER, El Jaouhari M, Eltayeb N, Abalos C, Striha M, Edjoc R, Ayoo C, Bonti-Ankomah S. Surveillance of laboratory exposures to human pathogens and toxins, Canada, 2021. Can Commun Dis Rep 2022;48(10):484–91. 10.14745/ccdr.v48i10a08 38125397 PMC10730106

[8] Atchessi N, Striha M, Edjoc R, Thompson E, El Jaouhari M, Heisz M. Surveillance of laboratory exposures to human pathogens and toxins, Canada 2020. Can Commun Dis Rep 2021;47(10):422–9. 10.14745/ccdr.v47i10a04 34737674 PMC8525605

[9] Lien A, Abalos C, Atchessi N, Edjoc R, Heisz M. Surveillance of laboratory exposures to human pathogens and toxins, Canada 2019. Can Commun Dis Rep 2020;46(9):292–8. 10.14745/ccdr.v46i09a07 PMC8525605 34737674

[10] Choucrallah D, Sarmiento L, Ettles S, Tanguay F, Heisz M, Falardeau E. Surveillance of laboratory exposures to human pathogens and toxins: Canada 2018. Can Commun Dis Rep 2019;45(9):244–51. 10.14745/ccdr.v45i09a04 31650987 PMC6781952

[11] Pomerleau-Normandin D, Heisz M, Tanguay F. Surveillance of laboratory exposures to human pathogens and toxins: Canada 2017. Can Commun Dis Rep 2018;44(11):297–304. 10.14745/ccdr.v44i11a05 30996692 PMC6449110

[12] Bienek A, Heisz M, Su M. Surveillance of laboratory exposures to human pathogens and toxins: Canada 2016. Can Commun Dis Rep 2017;43(11):228–35. 10.14745/ccdr.v43i11a04 29770052 PMC5764741

[13] El Jaouhari M, Striha M, Edjoc R, Bonti-Ankomah S. Laboratory-acquired infections in Canada from 2016 to 2021. Can Commun Dis Rep 2022;48(7-8):303–7. 10.14745/ccdr.v48i78a02 37334256 PMC10275617

[14] Wong C, Ng SY, Tan SH. An accidental laboratory exposure to *Brucella melitensis*: the prospective post-exposure management and a detailed investigation into the nature of the exposure. J Med Microbiol 2018;67(7):1012–6. 10.1099/jmm.0.000772 29846154

[15] Peng H, Bilal M, Iqbal HM. Improved Biosafety and Biosecurity Measures and/or Strategies to Tackle Laboratory-Acquired Infections and Related Risks. Int J Environ Res Public Health 2018;15(12):2697. 10.3390/ijerph15122697 30501091 PMC6313313

[16] Weng Choy K. Changes in clinical laboratory operations and biosafety measures to mitigate biohazard risks during the COVID-19 pandemic. The Lancet Microbe, 2020;1(7): e273–4, ISSN 2666-5247. 10.1016/S2666-5247(20)30168-3 10.1016/S2666-5247(20)30168-3 PMC7837007 33521724

[17] Public Health Agency of Canada. Canadian Biosafety Standard (CBS) Third Edition. Ottawa, ON: PHAC. [Accessed 2024 April 19]. https://www.canada.ca/en/public-health/services/canadian-biosafety-standards-guidelines/third-edition.html

